# Harbor Porpoise Deaths Associated with *Erysipelothrix rhusiopathiae*, the Netherlands, 2021

**DOI:** 10.3201/eid2904.221698

**Published:** 2023-04

**Authors:** Lonneke L. IJsseldijk, Lineke Begeman, Birgitta Duim, Andrea Gröne, Marja J.L. Kik, Mirjam D. Klijnstra, Jan Lakemeyer, Mardik F. Leopold, Bas B. Oude Munnink, Mariel ten Doeschate, Linde van Schalkwijk, Aldert Zomer, Linda van der Graaf-van Bloois, Els M. Broens

**Affiliations:** Utrecht University, Utrecht, the Netherlands (L.L. IJsseldijk, B. Duim, A. Gröne, M.J.L. Kik, J. Lakemeyer, M. ten Doeschate, L. van Schalkwijk, A. Zomer, L. van der Graaf-van Bloois, E.M. Broens);; Erasmus University Medical Center, Rotterdam, the Netherlands (L. Begeman, B.B. Oude Munnink);; Wageningen Food Safety Research, Wageningen, the Netherlands (M.D. Klijnstra);; Wageningen Marine Research, Den Helder, the Netherlands (M.F. Leopold)

**Keywords:** Erysipelothrix rhusiopathiae, cetaceans, zoonoses, strandings, pathology, bacteria, the Netherlands

## Abstract

In August 2021, a large-scale mortality event affected harbor porpoises (*Phocoena phocoena*) in the Netherlands. Pathology and ancillary testing of 22 animals indicated that the most likely cause of death was *Erysipelothrix rhusiopathiae* infection. This zoonotic agent poses a health hazard for cetaceans and possibly for persons handling cetacean carcasses.

*Erysipelothrix* bacteria cause infections in humans and other species after contact with infected animals or environmental sources (*1*). Illness ranges from mild to systemic, which can include septicemia and endocarditis. *Erysipelothrix* can survive for long periods in the environment, including marine ecosystems (*1*) associated with marine fish, mollusks, and crustaceans. *Erysipelothrix* infection affects captive and free-ranging crustaceans and is linked to fatal sepsis (*2*). To our knowledge, reports of large-scale mortality events caused by *Erysipelothrix* infection in marine mammals are absent from the literature, and *Erysipelothrix* has not been detected in stranded porpoises along the Netherlands coastline since the start of our harbor porpoise stranding research program in 2008.

At the end of August 2021, a total of 190 dead harbor porpoises (*Phocoena phocoena*) were found on Dutch Wadden islands; the annual average for stranded harbor porpoises on the entire Dutch coastline is 600. No anthropogenic activities in the southern or central North Sea that could explain this mortality event were reported to the government of the Netherlands in the 4–6 weeks before the event.

Most porpoises were found in an advanced state of decomposition. Twenty-two animals were collected for examination at the Faculty of Veterinary Medicine of Utrecht University (Appendix Table 1). We immediately necropsied 2, and the rest were temporarily frozen pending postmortem investigation and ancillary testing.

Because of advanced decomposition, we could perform only gross pathologic examinations and sampling for ancillary testing, following a standardized international protocol (*3*). Adult female porpoises were mostly in good to moderate nutritional condition with mild to moderate parasitic infections of various organs and had been reproductively active (Appendix Table 1). Of the 21 stomachs examined (1 was not examined because of gross damage caused by scavengers), none contained marine debris; 10 contained the remains of a few prey, reflecting nonrecent food intake, and the remaining stomachs were empty.

Samples from 3 porpoises with gross changes (mammary gland, lung, spinal cord) were cultured on blood agar (bioTrading, https://biotrading.com) at 37°C for 48 h. Culture results were positive for *Erysipelothrix rhusiopathiae*. Subsequently, we tested liver samples from 21 animals for *E. rhusiopathiae*; and 16 were positive (Appendix Table 2). To investigate the relatedness of isolates, genomes of 18 isolates were sequenced by using Illumina NextSeq (https://www.illumina.com) and assembled by using SPAdes version 3.14.1 (*4*); we included 11 publicly available reference genomes from different *E. rhusiopathiae* clades (*5*). A core genome alignment was made with Parsnp verson 1.2 (*6*) and visualized by using iTol version 4 (*7*).

Genomes from this study were phylogenetically positioned between clade 2 *E. rhusiopathiae* reference genomes and formed 2 distinct clusters showing ≈3,400 single-nucleotide polymorphism (SNP) differences and limited diversity of <6 SNPs within the clusters (Figure). That pattern suggests dissemination of 2 clonal lineages of *E. rhusiopathiae*, either through exposure to a common source or contact between individuals.

**Figure:**
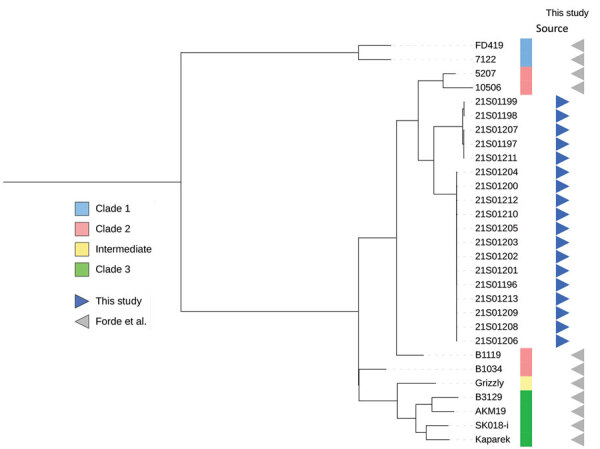
Phylogenetic tree of *Erysipelothrix rhusiopathiae* from stranded harbor porpoises, the Netherlands, 2021, compared with reference genomes described by Forde et al. (*5*). Branches are square root transformed. Detailed information for each sample is provided in Appendix Table 3.

Virology tests on 14 fecal, 15 blood, and 17 spleen samples and metagenomic sequencing with VirCapSeq-VERT (*8*) revealed no virus sequences of interest. In addition, we tested 20 lung and 20 brain samples for influenza A virus, paramyxoviruses (including morbilliviruses), coronaviruses (including SARS-CoV-2), and herpesviruses. Only 2 brain samples tested positive for *P. phocoena* alphaherpesvirus (Appendix Table 1), described as an incidental cause of death in porpoises (*9*). Our results indicate that viruses were an unlikely factor in this mortality event.

We pooled 20 stomach content samples and 21 liver samples in triplicate and analyzed them with a Liquid Chromatograph Triple Quadrupole Mass Spectrometer (LC-MS/MS) (McCrone Associates, https://www.mccrone.com) for domoic acid, saxitoxins, tetrodotoxin, and lipophilic marine toxins. Only saxitoxin was detected; it was in 1 pooled liver sample (estimated concentration 15 μg/kg). Subsequently, we analyzed livers individually, and saxitoxin was not confirmed in any of the individual samples. We therefore conclude that harmful algae were an unlikely factor in this mortality event.

Gross pathologic assessment revealed a moderate to good body condition for most porpoises, but none had recently fed. This finding suggests a subacute cause of death from sudden and excessive disease. No clinically relevant viruses were detected. Phycotoxins were detected in a limited number of porpoises. In contrast, *E. rhusiopathiae* was isolated from most investigated porpoises. Therefore, we consider *E. rhusiopathiae* to be the most likely cause of death. Advanced autolysis of the carcasses made detection of distinctive lesions associated with *Erysipelothrix* infection impossible. The low number of SNPs differing between isolates suggests common exposure, possibly a food source, transmission between porpoises, or both.

Our results draw attention to possibly increased cetacean susceptibility to *E. rhusiopathiae*, to new or emerging sources of *Erysipelothrix* in the marine environment, or both. *Erysipelothrix* remains viable in a carcass up to 12 days in direct sunlight, up to 4 months in putrefied flesh, and up to 9 months in a buried carcass (*10*). This new emerging source and the long survival time in carcasses demonstrates a need for having only trained personnel handle stranded animals, proper disposal of carcasses, and increased awareness for the potential presence and transmission of this zoonotic bacterium among cetaceans.

Appendix Supplementary results from study of harbor porpoise deaths associated with *Erysipelothrix rhusiopathiae*, the Netherlands, 2021.
