# Supplementary material for: Harbor Porpoise Deaths Associated with Erysipelothrix rhusiopathiae, the Netherlands, 2021
Source: Emerg Infect Dis. 2023 Apr;29(4):835–8. doi: 10.3201/eid2904.221698 (PMC10045706; doi:10.3201/eid2904.221698)
Supplement: Appendix — Supplementary results from study of harbor porpoise deaths associated with Erysipelothrix rhusiopathiae, the Netherlands, 2021. [file 22-1698-Techapp-s1.pdf]

Article DOI: https://doi.org/10.3201/eid2904.221698

EID cannot ensure accessibility for supplementary materials supplied by authors. Readers who have difficulty accessing supplementary content should contact the authors for assistance.

Harbor Porpoise Deaths Associated with *Erysipelothrix rhusiopathiae*, the Netherlands, 2021

Appendix

Appendix Table 1. Overview of biological, pathological and relevant ancillary testing data of investigated harbor porpoises*

Case ID	Stranding date	Stranding location	Age	Sex	DCC	NCC	Mass (kg)	TL (cm)	Repro assessment	Erysipelothrix	Phocoena phocoena alphaherpesvirus	Phycotoxins
UT1877	23-8-2021	Schiermonnikoog	A	M	3	5	27	130	Adult male	positive	NA	Pooled samples stomach and liver negative
UT1878	26-8-2021	Schiermonnikoog	A	F	4	5	35	154	Large CL but no embryo detected, suspect aborted	positive	NA	
UT1880	28-8-2021	Ameland	A	M	4	3	32	138	Adult male	positive	negative	
UT1881	28-8-2021	Ameland	J	F	4	2	19	122	Juvenile female	positive	positive (brain)	
UT1882	28-8-2021	Ameland	A	F	4	2	23	136	Scavenged	positive	negative	
UT1883	28-8-2021	Ameland	A	F	4	3	44	157	Scavenged	positive	negative	
UT1884	28-8-2021	Ameland	A	F	4	2	42	153	Lactating and pregnant	positive	negative	
UT1885	28-8-2021	Ameland	A	F	4	1	35	144	Pregnant	negative	negative	Pooled samples stomach and liver negative
UT1886	28-8-2021	Ameland	A	M	4	6	22	141	Adult male	positive	negative	
UT1887	28-8-2021	Ameland	A	F	4	1	41	153	Lactating, extended uterus, no embryo detected	positive	positive (brain)	
UT1888	28-8-2021	Ameland	A	F	4	1	32	153	Cervix extended, further scavenged hampering assessment	positive	negative	
UT1889	28-8-2021	Ameland	A	M	4	5	29	134	Adult male	negative	negative	
UT1890	28-8-2021	Ameland	A	F	4	4	34	152	Lactating, CL left, no embryo detected	positive	negative	
UT1891	28-8-2021	Schiermonnikoog	A	F	4	1	51	165	Pregnant	negative	negative	
UT1892	27-8-2021	Schiermonnikoog	A	F	4	1	44	154	Resting	negative	negative	Pooled sample stomach negative; pooled samples liver positive for
UT1893	28-8-2021	Terschelling	A	F	4	6	44	163	Lactating and pregnant	positive	negative	
UT1894	28-8-2021	Terschelling	A	F	4	2	27	147	Lactating and pregnant	positive	negative	
UT1895	28-8-2021	Terschelling	A	F	4	4	33	152	Pregnant	positive	negative	
UT1896	28-8-2021	Terschelling	A	F	4	4	35	164	Resting	positive	negative	

Case ID	Stranding date	Stranding location	Age	Sex	DCC	NCC	Mass (kg)	TL (cm)	Repro assessment	Erysipelothrix	Phocoena phocoena alphaherpesvirus	Phycotoxins
UT1897	28-8-2021	Terschelling	A	F	4	2	44	162	Lactating and pregnant	positive	negative	SXT (15 µg/kg)
UT1898	28-8-2021	Terschelling	A	F	4	3	37	163	Scavenged	negative	negative	
UT1899	28-8-2021	Terschelling	J	M	4	4	13	114	Juvenile male	NA	negative	NA

*Dates are DD-MM-YYYY. Age: A = adult, J = Juvenile; Sex: M = Male, F = Female; DCC: Decomposition Condition Code, following L.L. IJsseldijk et al., unpub. data,https://eprints.gla.ac.uk/270811; NCC: Nutritional Condition Code, following L.L. IJsseldijk et al., unpub. data. NA = Not Available; STX = saxitoxins

Appendix Table 2. Detailed results of bacteriological culture

	Bacteriology	
ID	Cultured organs	Culture results
UT1877	Liver	Liver: 1. Mild amount of Proteus mirabilis, 2. Moderate amount of E. rhusiopathiae
UT1878	Liver and lung	Liver: Moderate amount of E. rhusiopathiae. Lung: 1. Severe amount of Clostridium perfringens, 2. Severe amount of E. rhusiopathiae, 3. Mixed culture of 3 bacteria, mild amount
UT1880	Liver	Liver: Moderate amount of E. rhusiopathiae
UT1881	Liver	Liver: 1. Mild amount of E. rhusiopathiae, 2. Mixculture of 2 bacteria, mild amount
UT1882	Liver	Liver: 1. Mild amount of E. rhusiopathiae, 2. Mild amount of anaerob mixed culture
UT1883	Liver	Liver: Moderate amount of E. rhusiopathiae
UT1884	Liver	Liver: 1. Moderate amount of E. rhusiopathiae, 2. Mild amount of mixed culture of 2 bacteria
UT1885	Liver	Liver: Mild amount of anaerob mixed culture
UT1886	Liver	Liver: 1. Moderate amount of E. rhusiopathiae, 2. Mild amount of anaerob mixed culture
UT1887	Liver	Liver: 1. Moderate amount of E. rhusiopathiae, 2. Mild amount of anaerob mixed culture
UT1888	Liver	Liver: 1. Moderate amount of E. rhusiopathiae, 2. Mild amount of anaerob mixed culture
UT1889	Liver	Liver: Mild amount of anaerob mixed culture
UT1890	Liver	Liver: Moderate amount of E. rhusiopathiae
UT1891	Liver	Liver: Mild amount of anaerob mixed culture
UT1892	Liver	Liver: Mild amount of anaerob mixed culture
UT1893	Liver	Liver: 1. Moderate amount of E. rhusiopathiae, 2. Mild amount of anaerob mixed culture
UT1894	Liver and mammary gland	Liver: Mild amount of Paeniclostridium sordelli. Mammary gland: 1. Moderate amount of E. rhusiopathiae, 2. Mixed culture of 2 bacteria
UT1895	Liver and spinal cord (swab)	Liver: 1. Mild amount of anaerob mixed culture, 2. Mild amount of E. rhusiopathiae. Swab CNS: 1. Moderate amount of E. rhusiopathia, 2. Mixed culture.
UT1896	Liver	Liver: 1. Moderate amount of E. rhusiopathiae, 2. Mild amount of anaerob mixed culture
UT1897	Liver	Liver: Moderate amount of E. rhusiopathiae
UT1898	Liver	Liver: no bacteria cultured
UT1899	NA	NA

NA, not available

Appendix Table 3. Detailed information for each sample

ASSEMBLY_NAME	VMDC_ID	ASSEMBLY_ACC	STUDY_ID	SAMPLE_ID
21S01196	221083103101	GCA_947039675	PRJEB56391	ERS13511510
21S01197	221093006801	GCA_947041805	PRJEB56391	ERS13511511
21S01198	221093006701	GCA_947037435	PRJEB56391	ERS13511512
21S01199	221101910801	GCA_947040135	PRJEB56391	ERS13511513
21S01200	221101911001	GCA_947037175	PRJEB56391	ERS13511514
21S01201	221101911101	GCA_947037165	PRJEB56391	ERS13511515
21S01202	221101911201	GCA_947039825	PRJEB56391	ERS13511516
21S01203	221101911301	GCA_947038085	PRJEB56391	ERS13511517
21S01204	221101911401	GCA_947040235	PRJEB56391	ERS13511518
21S01205	221101911601	GCA_947037575	PRJEB56391	ERS13511519
21S01206	221101911801	GCA_947040195	PRJEB56391	ERS13511520
21S01207	221101911901	GCA_947038325	PRJEB56391	ERS13511521
21S01208	221101912101	GCA_947038715	PRJEB56391	ERS13511522
21S01209	221101912401	GCA_947038205	PRJEB56391	ERS13511523
21S01210	221101912601	GCA_947038705	PRJEB56391	ERS13511524
21S01211	221101912701	GCA_947037675	PRJEB56391	ERS13511525
21S01212	221101912801	GCA_947038755	PRJEB56391	ERS13511526
21S01213	221101910901	GCA_947038735	PRJEB56391	ERS13511527
